# NOSACQ-50 for Safety Climate Assessment in Agricultural Activities: A Case Study in Central Italy

**DOI:** 10.3390/ijerph17249177

**Published:** 2020-12-08

**Authors:** Mario Fargnoli, Mara Lombardi

**Affiliations:** Department of Chemical Engineering Materials Environment (DICMA), Sapienza-University of Rome, via Eudossiana 18, 00184 Rome, Italy; mara.lombardi@uniroma1.it

**Keywords:** safety climate, NOSACQ-50 questionnaire, occupational health and safety, safety behavior, risk perception, agriculture, safety culture, sustainable agricultural systems, ergonomics, human factors

## Abstract

Safety climate assessment has been recognized as a powerful tool to bring to light workers’ perceptions related to safety practices, attitudes and behaviors at the workplace. Accordingly, its investigation can provide useful information about safety problems within a company before accidents occur. In literature, numerous studies can be found investigating safety climates in different types of industry. However, safety climate assessment in agricultural activities is addressed scarcely, even though agriculture represents a very hazardous sector. To reduce this gap, the present study proposes an investigation of safety climate among farmers by means of the Nordic Safety Climate Questionnaire (NOSACQ-50). The results of the survey brought to light a low level of safety perception of the interviewed sample, especially as concerns safety commitment and risk-taking attitudes. In particular, a different attitude toward safety issues has been registered depending on both the position and gender of the interviewed farmers. Overall, the output of this cross-sectional analysis adds to the current literature by expanding knowledge of safety climate among farmers, representing a baseline for further investigations based on human-centered approaches to enhance safety in agriculture and consequently social and workplace aspects of sustainable agricultural systems.

## 1. Introduction

Occupational health and safety (OHS) issues represent key factors of the social pillar of sustainable development: accordingly, safety-related initiatives to reduce occupational accidents and illnesses support the operationalization of sustainability within companies [1]. In such a context, the evaluation of safety climate (SC) has been considered a powerful research approach [2], since its assessment is regarded as a means to collect information about safety problems practically before they lead to workers’ accidents [3]. In other words, as argued by Seo et al. [4], SC investigation can bring to light organizational and cultural factors that are precursors of accidents. Actually, these authors stressed the fact that the main benefit of assessing workers’ SC relies on its association with safety practices, accidents and unsafe behaviors. Accordingly, SC assessment provides information on the workers’ perception of safety in their work environment [5], resulting in an effective tool for evaluating their safety performances in a specific context [6,7,8]. Hence, it is deemed that investigating SC represents a crucial factor for the prevention of accidents and the improvement of the level of safety at the workplace [9]. SC indicates the workers’ perception of safety policies, procedures, and practices, which influences workers’ safety behavior and attitude largely in a specific working and environmental context [10]. In fact, as argued by Pandit et al. 2019 [11], a negative SC contributes to a low level of both hazard recognition and safety risk perception. Consequently, several approaches for the assessment of SC have been proposed [4,12,13,14], which are mostly based on the investigation of the six dimensions of SC established by Zohar [10] relying on the workers’ perception of different aspects, ranging from management attitudes towards safety, to the importance and effectiveness of safety training, and the effectiveness of enforcement versus guidance in promoting safety.

Therefore, given the importance of focusing on human-centered approaches to enhance safety management [15], numerous studies have investigated SC in different types of industry [2], for example the construction sector [16,17], as well as the mining, oil and gas extraction [8,18,19], healthcare [20], fire service [21], and aviation [22] sectors. However, when it comes to agricultural activities, very little research can be found addressing SC, although agriculture is deemed to be a very hazardous working environment [23,24,25,26]. To deal with such a phenomenon, increasingly severe standards and regulations concerning occupational safety have been introduced [27,28]. Nevertheless, the number of accidents and victims has not significantly decreased: for instance, according to data published by the Italian Compensation Authority (INAIL) in 2019 the number of fatal accidents which occurred in the agriculture sector increased by 15% (151 fatalities versus 131 registered in 2018) [29]. In addition, also professional diseases among farmers are increasing (+1.9% from 2018 to 2019): in particular, an increase of 2.3% of musculoskeletal pathologies related to biomechanical overload and repeated movements was registered, which can also be considered an index of poor safety attitudes and practices.

The growing awareness of the importance of safety in the agricultural activities has also led to an increasing amount of research, mostly focusing on the implementation of technical solutions aimed at improving the safety level of farmers [30,31]. Conversely, the analysis of work behavior and attitudes relevant to safety has not been addressed sufficiently, as stressed by Irwin and Poots [32]. The authors highlight that although psychosocial factors have a negative impact on the safety performance of farmers, little research has been conducted to investigate their attitudes toward safety. Similarly, other studies have demonstrated that having a low-risk perception can lead to an augmented exposure of farmers to occupational risks and accidents [33,34]. Caffaro et al. [35] also noted that neglecting the importance of safety laws and regulations represents an important risk factor for accidents as this can legitimate safety non-compliance. Moreover, this situation is particularly critical for small and medium-sized enterprises, which characterize the majority operating in this sector and where the implementation of safety management solutions results in being more difficult than in larger-sized organizations [36,37,38]. Hence, the evaluation of SC among small-sized farms can augment knowledge on safety management in this specific context, contributing to identifying workplace dynamics and behaviors related to safety that can be used to implement effective measures at the procedural and normative levels to augment safety attitude and practices.

Based on the above considerations, this study aims at reducing the scarcity of research on SC in agriculture. With this goal in mind, the Nordic Safety Climate Questionnaire (NOSACQ-50) [39] was used to carry out a survey among farmers in the Lazio region (central Italy). The results achieved allowed us to analyze SC within these companies, screening the different perceptions of workers and managers, as well as those that emerged based on the gender and age of the interviewed farmers.

The remainder of the article is organized as follows: in the next section, a background analysis is provided addressing previous research on SC in the agriculture sector to support our research motivations. Then, in Section 3 material and methods are described, while Section 4 illustrates the results of the survey. Finally, the discussion of the results is presented in Section 5 and the conclusive remarks are provided in Section 6.

## 2. Background Analysis

### 2.1. Safety Climate Assessment in Agriculture

To better understand the research trends and issues on SC in the agriculture sector, a literature review was carried out searching in the scientific database Scopus. More in detail, following the approach proposed by several authors [40,41,42], the following criteria were used in the analysis:

- Database: Scopus;
- Search string: TITLE-ABS-KEY (“safety climate” AND (“agricultur*” OR “farm*”));
- Period: no limit;
- Source type: journal articles published in English;
- Eligibility: full-text analysis for the inclusion of those articles dealing with the assessment of SC among agricultural workers only.

Overall, 29 articles were found. Then, a further filtering was carried out based on the eligibility criteria: in other words, the selected documents were analyzed to distinguish those whose main goal consisted in evaluating SC among farmers from the others. For instance, among the 29 documents provided by Scopus, those focusing on food safety and climate were excluded. Similarly, a study of SC among office workers at the Department of Agriculture was not included, as well as those articles dealing with general surveys on the quality of work life and risk factors. As a result, 17 documents were selected published from 2004 to 2020, whose main features are summarized in Table 1.

Examining the text of the above documents, it emerged that most of them consist in community-based analyses, aimed at investigating SC in relation to other aspects such as: musculoskeletal discomfort, depression and stress. In fact, they took into account specific groups of farmers especially among migrants, who have more problems in dealing with occupational issues because of difficulties related to language, education level, and habits [46,47,49,50,51,52,53]. Other studies analyzed SC among operators in specific agricultural activities, such as aquaculture [44], grain elevators’ management [4], and viticulture [45].

From a methodical point of view, most studies adapted the Perceived Safety Climate (PSC) proposed by Gillen et al. [58] to their specific contexts. Such a tool was developed in the construction industry context and it is aimed at investigating the following 10 main aspects:

1. Workers’ safety practices are very important to management;
2. Workers are regularly made aware of dangerous work practices or conditions;
3. Workers have almost total control over personal safety;
4. Workers receive instructions on safety when hired;
5. Proper safety equipment is always available;
6. Taking risks is not a part of my job;
7. Workers attend regular safety meetings;
8. Workers are regularly praised for safe conduct;
9. The possibility of being injured at work in the next 12 months is very likely;
10. Supervisors seem to care about your safety.

By contrast, Grimbuhler and Viel [45] developed a specific SC questionnaire aimed at investigating the implementation of best practices when applying pesticides in vineyards, due to the peculiarities and specific risks related to this type of activity [59]. This questionnaire consists of 20 items grouped into the following seven main SC aspects:

1. Management commitment;
2. Communication and feedback;
3. Rules and best practices;
4. Knowledge;
5. Safety compliance;
6. Safety participation;
7. Teamwork climate.

In a similar manner, Kongsvik et al. [44] implemented a specific SC questionnaire based on previous studies in the maritime domain, which consists of 17 items (statements). Wilmsen et al. [46] developed a specific SC questionnaire based on a set of questions that take into account the following main aspects: training, safety meetings, rest breaks, site inspections. Based on the approach proposed by Neal et al. [60] within a hospital setting, Irwin and Poots [32] used a SC questionnaire aimed at investigating the following three main aspects: safety motivation, safety compliance, and risk tolerance. The study of Neal et al. [60] was used as a basis by Savadori et al. [54] as well: in this case, the focus of the analysis was safety compliance. Finally, it is worth mentioning that Seo et al. [4] adopted a SC questionnaire based on 32 items considering the following issues:

1. management commitment,
2. supervisor support,
3. coworker support,
4. employee participation,
5. competence level.

As far as the practical assessment of the above items is concerned, the use of a Likert scale to state the level of agreement of the respondents was the common method in the selected studies, usually adopting a 5-point scale ranging from 1 (never) to 5 (always) in case of direct questions or 1 (totally disagree) to 5 (totally agree) in case of statements. Furthermore, it is worth mentioning that the analysis of psychological elements, such as the subjective assessment of safety that can be derived by the SC measurement, contributes to determine also the safety culture status among the interviewed workers [17,61]. Based on this, echoing Westaby and Lee [62], Arcury and colleagues [43,53] have investigated SC among different groups of farmers to understand their work safety culture level, which encompasses behavioral, situational, and psychological elements of safety.

Finally, in order to evaluate the impact in literature of these studies compared to those addressing SC in other sectors, two additional searches in Scopus were carried out considering the construction and manufacturing industries:

- In the construction industry: search string: TITLE-ABS-KEY (“safety climate” AND (“construction” OR “building”)) AND (LIMIT-TO (LANGUAGE, “English”) AND (LIMIT-TO (SRCTYPE, “j”)); output: 266 studies.
- In the manufacturing and operation sector: search string: TITLE-ABS-KEY (“safety climate” AND (“manufactur*” OR “operation*”)) AND (LIMIT-TO (LANGUAGE, “English”)) AND (LIMIT-TO (SRCTYPE, “j”)); output: 175 studies.

Thus, although one might correctly argue that 29 documents discussing SC in agriculture is a substantial number in general, when comparing it with those obtained in different types of sector, this number appears to be small, showing that SC among farmers is rarely investigated.

### 2.2. Research Issues

Based on the above considerations, it is deemed that the assessment of SC among farmers is scarcely addressed if compared to other sectors [14,57], while its assessment can lead to undercover psychological measures that are related to the occurrence of injuries [51]. Moreover, the extant literature focuses mainly on the SC investigation among workers belonging to minorities and vulnerable groups within the same sector (i.e., case-control studies), or on the analysis of the relationship between SC and specific health problems, limiting the research findings to that specific context only. Conversely, the lack of cross-sectional analyses depicting the current situation among farmers is pointed out [44,45]. Moreover, it has to be noted that small-sized and family-run companies have difficulties in implementing organizational and management solutions aimed at improving the safety at workplace [63,64], such as SC monitoring [65], although their situation with respect to health and safety is worse than that of larger companies, since most accidents occur on small farms [54].

At the same time, it was also observed that different research approaches were followed to measure SC, making the comparison and extension of these studies more difficult for both researchers and practitioners. Differently, the use of an established tool such as NOSACQ-50, which has been validated in various contexts, can allow for a larger usability of data [40]. Nonetheless, a cross-sectional investigation of SC among farmers by means of the Nordic questionnaire is still missing in literature. Hence, to reduce the aforementioned gaps, this study aims at augmenting knowledge on SC in agriculture through the application of NOSACQ-50.

## 3. Materials and Methods

NOSACQ-50 was presented by Kines et al. [66] in 2011 as the result of research activities involving a Nordic network of occupational safety researchers and it was available online [39]. The reliability and validity of NOSACQ-50 has been tested in various studies in different contexts, confirming its effectiveness as a diagnostic tool to investigate the safety climate within organizations [67,68,69]. The questionnaire consists of 50 statements, which contribute to evaluate seven SC dimensions. In detail, the aggregation of the statements for each dimension is provided as follows:

- Dim1—Management safety priority, commitment, and competence: 9 statements to evaluate workers’ perception of safety management.
- Dim2—Management safety empowerment: 7 statements to evaluate workers’ perception of management empowerment and support to participate in safety issues.
- Dim3—Management safety justice: 6 statements addressed to estimate how workers perceive accidents’ management.
- Dim4—Workers’ safety commitment: 6 statements to indicate how workers perceive their own commitment to safety.
- Dim5—Workers’ safety priority and risk non-acceptance: 7 statements indicating the workers’ risk-taking attitude and safety prioritization in their working tasks.
- Dim6—Safety communication, learning, and trust in co-workers safety competence: 8 statements investigating how workers perceive the exchange of safety knowledge and experiences among themselves.
- Dim7—Trust in the efficacy of safety systems: 7 statements to analyze workers’ perception of benefits derived from safety planning, training, monitoring, etc.

As far as the assessment criteria are concerned, a four-step Likert scale is used for rating each statement using the terms strongly disagree, disagree, agree, and strongly agree, which namely correspond to a 1–4 rating scale in case of positively formulated statements or 4–1 for the reversed statements. Accordingly, as suggested in the NOSACQ-50 website [39], which is managed by the Division of Safety Research of the National Research Centre for the Working Environment of Denmark, the evaluation of each dimension is based on the criteria summarized in Table 2, providing an easy-to-use reference for the interpretation of the questionnaire results [66]. More in detail, in the questionnaire, interviewed farmers were asked to indicate for each of the 50 statements an agreement/disagreement evaluation selecting one of the four options: strongly disagree, disagree, agree, and strongly agree. Then, depending on the type of statement (positively or reversed) the score was assigned as indicated above.

**Figure 1:**
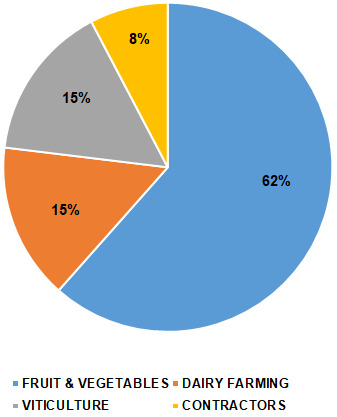
Specific activities/working sectors of the interviewed companies.

In addition, background information is also requested in the questionnaire, concerning age, gender, and position within the company (i.e., workers and managers). The interviews in this study were carried out by self-administered questionnaires to 49 companies involved in agricultural activities in the Italian region of Lazio, which accounts for about 5000 companies in the agriculture sector. A farmers’ union provided us with a list of the companies we asked to collaborate with: among them, 35 companies responded positively (71% of the contacted companies) and a total number of 108 responses, including both managers and workers, was collected. It has to be noted that all the companies are small-sized or family-run enterprises, whose specific activities range from fruit and vegetables cultivation to viticulture and dairy farming (Figure 1).

Due to the particular period during which the interviews were carried out (spring season and COVID-19 restrictions) no seasonal or foreign workers were found in the sample and all the interviewed workers were regularly employed. All the companies were contacted personally, and a meeting to explain the objectives of the study and the NOSACQ-50 features was carried out. At each company, the questionnaires were filled in simultaneously by both workers and managers, and collected together to avoid any bias and to guarantee the anonymity and privacy of farmers in accordance with Italian law.

## 4. Results

Results related to the background information of the interviewed sample are reported in Table 3 where data on the age, gender and position of the farmers are summarized (it has to be noted that 2 people did not provide their age, while 1 person did not indicate the position).

**Figure 2:**
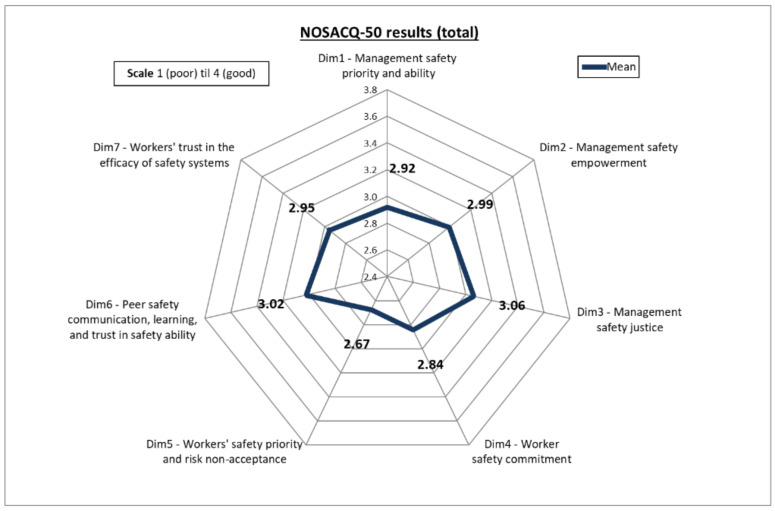
Results of the NOSACQ-50 questionnaire related to all the interviewed farmers.

The output of the questionnaires was obtained following the NOSACQ-50 approach, which provides a mean score for each of the seven SC dimensions. In Figure 2 the results related to all the interviewed people (both managers and workers) are reported. From these data, it emerges that not all the seven SC dimensions have a good level: only peer safety communication (Dim6) and management safety justice (Dim3) require a slight improvement, while most dimensions (Dim1, Dim2, Dim3 and Dim7) need a certain improvement and Dim5 (workers’ safety priority and risk non-acceptance) requires great improvement.

Analyzing the data of the case study more in detail, substantial differences between different groups within the interviewed sample emerged, as shown it Table 4 where the results based on the position (managers and workers) and gender (males and females) are reported.

**Figure 3:**
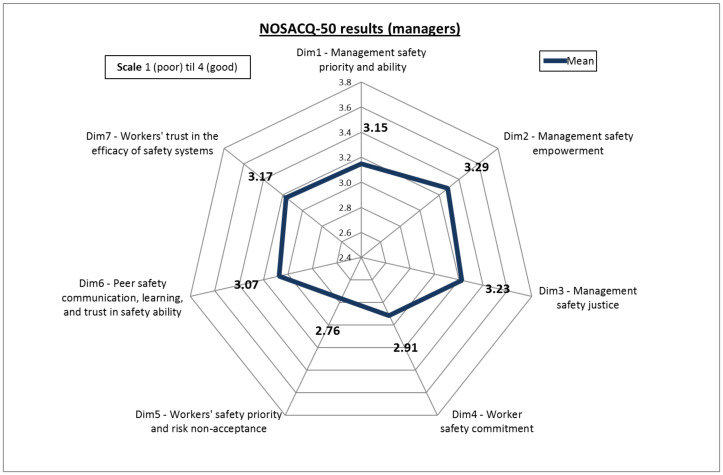
Overall scores obtained by managers.

**Figure 4:**
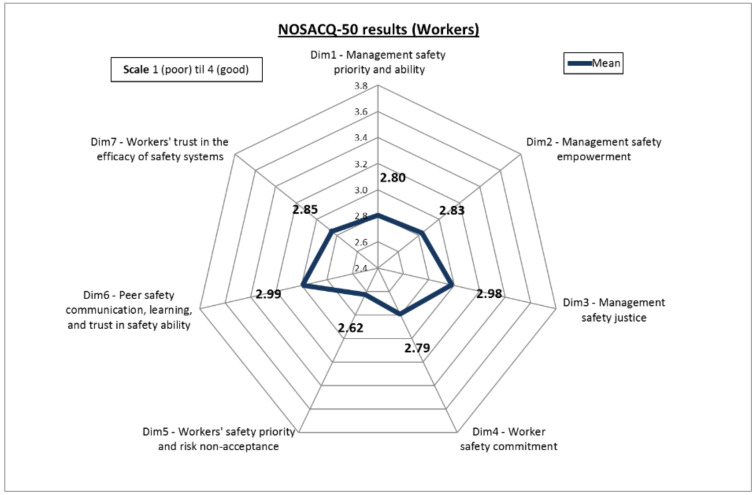
Overall scores obtained by workers.

Given that in NOSACQ-50 the first three dimensions concern the perceptions of the organizational safety of the company while the other four are more focused on the workers’ safety management, all groups in our sample showed a higher score for the organizational level dimensions (Dim1, Dim2 and Dim3). More in detail, screening the results based on the position within the organization, the scores obtained show a higher level of SC among managers (Figure 3) compared to those scored by workers (Figure 4). In particular, managers obtained “fairly good” results not only for organizational dimensions but also for Dim6 (Safety communication, learning, and trust in co-workers safety competence) and Dim7 (Trust in the efficacy of safety systems).

**Figure 5:**
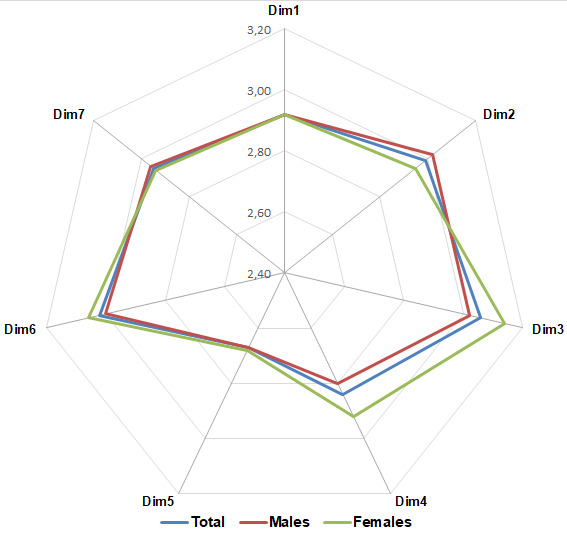
Overall scores obtained by males and females compared to the total values obtained by the interviewed sample.

Another interesting result is related to the discrepancies that emerged between males and females: as shown in Figure 5, they concern both the general trend of the SC perception (compared to the overall pattern) and the differences in score related to specific SC dimensions.

In particular, differences between males and females concern: Dim2 (management safety empowerment); Dim3 (management safety justice); and Dim4 (workers’ safety commitment).

**Figure 6:**
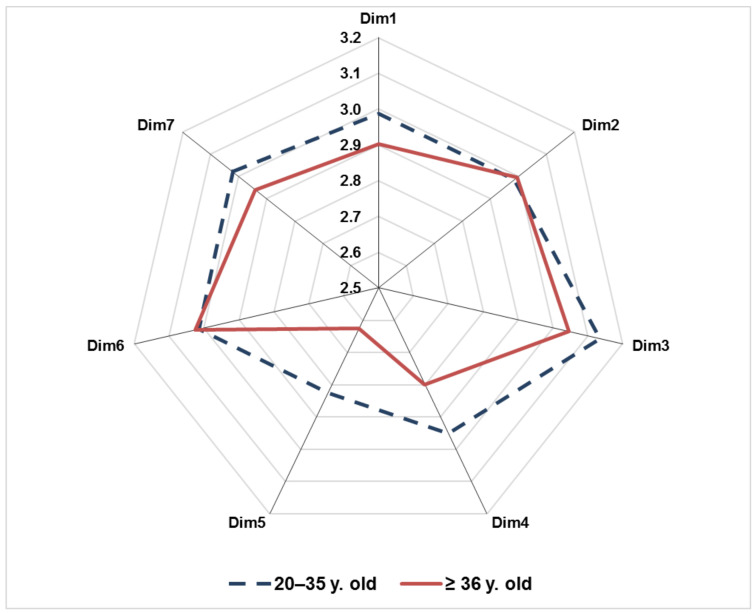
Scores obtained by farmers differentiated based on the age (workers 20–35 years old, dotted line; workers older than 35 year old, solid line).

Then, a further analysis based on the respondent’s age was also carried out differentiating between farmers of 20–35 and older ones. As shown in Figure 6, the scores of SC dimensions are mostly higher for younger farmers than those for aged farmers, demonstrating a lower SC perception of the latter.

Finally, in order to verify if inferential relationships exist in these groups statistically, the t-test was performed, considering the level of significance at *p* < 0.05 [14,70] (all analyses were made using MS Excel@ 2016 software, Microsoft, Redmond, Washington DC, USA). The output of these analyses is reported in Table 5.

These results show that while the first subdivision of the sample (managers-workers) statistically impacts on the SC values. Conversely, in the case of groups subdivided based on the differences of gender and age these differences are not statistically significant in terms of SC perception.

## 5. Discussion of Results

### 5.1. Case Study Outcomes

The study provides an exploratory and descriptive analysis of SC perception among farmers of small-sized or family-run companies. Looking at the overall results, the fact that most SC dimensions need improvement brings to light a low level of safety perception of the interviewed sample at their workplace. This is a not surprising result, since such an aspect confirms the assumption that a low level of SC is related to a high rate of occupational injuries and diseases [71], and numerous recent studies have shown the vulnerability of farmers to occupational accidents [31,72,73]. In particular, consistent with other studies [14,74], overall values showed a higher score for the organizational-level dimensions (mean value for Dim1, Dim2, and Dim3 = 2.99) than that related to the worker-level climate (mean value for Dim4, Dim5, Dim 6, Dim7 = 2.87).

More interestingly, despite the aforementioned differences, a similar pattern of the scores obtained by managers and workers respectively has to be pointed out. This finding fits well with the characteristics of the companies of our sample, which are small and very small enterprises (in some cases only two people: one manager and one worker). This feature is very common in the agriculture sector in Italy and in this type of company managers carry out similar working activities, sharing the same risk-exposures and working conditions. Such an aspect can be considered similar to the results achieved by Marin et al. [14] when comparing the SC scores of supervisors and workers in the construction sector. Accordingly, it is not surprising that the differences in the score for the SC dimensions are more related to in-field daily activities (i.e., Dim4, Dim5, and Dim6) are smaller than others. For instance, with regard to Dim6, which evaluates how workers perceive the exchange of safety knowledge and experiences, the discrepancy is very slight (2%). This might be attributable to the fact that in the companies of our sample managers play the double role of administrators and workers at the same time. Hence, they are aware of the risk exposure in a similar manner that workers are. This finding also confirms the similarities between these two sectors (agriculture and construction) when it comes to occupational safety issues in small-sized companies [74,75,76]. Conversely, the managers’ higher perception of SC dimensions, especially those related to organizational issues (Dim1, Dim2, Dim3), can be explained by the usual role of owner-manager characterizing small enterprises [63], which lead managers to overestimate their safety management abilities enhancing the company’s image [14].

Another interesting aspect is related to the discrepancies that emerged between males and females, which concern both the general trend of the SC perception (compared to the overall pattern) and the differences in score related to specific SC dimensions.

In fact, most noteworthy differences concern Dim2 (females perceived a lower level of management safety empowerment), Dim3 (males have a lower perception of justice related to the management of occupational accidents), and Dim4 (females have a higher perception of their commitment to safety). Additionally, it appears that males have more problems in safety communication (Dim6). Apart from psychological/behavioral issues related to gender differences that are not the object of the present study, the above results can be partially explained taking into account the fact that men are usually more exposed to serious injuries since they are much more involved in hazardous activities (e.g., the use of tractors) and do more physically strenuous jobs than women. Moreover, these findings support the research by Gyekye and Salminen [77], who stressed on the higher safety perceptions of female industrial workers compared to male co-workers.

Conversely, both the scores related to Dim1 (workers’ perception of safety management) and Dim5 (workers’ risk-taking attitude and safety prioritization in their working tasks) are similar for males and females. This implies that the general low level of these SC dimensions is perceived in the company homogenously.

Some notable differences emerged taking into account the differences between young and aged farmers:

- Younger workers have a higher perception of safety issues especially for what concerns Dim1 (management safety priority and ability), Dim 4 (workers’ safety commitment), and Dim5 (workers’ safety priority and risk non-acceptance). This aspect can be related to a higher level of awareness on safety matters due to a higher level of information/education. In fact, although a specific analysis of the education level was not conducted, such an assumption can be derived from the preliminary meetings carried out with the respondents. Moreover, this is supported by recent studies [78] and data provided by the Italian Ministry of agriculture/National Rural Network [79].
- Conversely, the lower perception of Dim5 (workers’ safety priority and risk non-acceptance) among older workers shows their high risk-taking attitude. Such a result shows that more experienced workers are prone to unsafe and risk-taking behavior, consistently with other studies in different sectors [80,81].

Overall, a different SC pattern emerged between these two subgroups, showing a lower SC perception of aged workers at a general level. On the one hand, this is in line with other studies underlining the fact that elder workers might be overconfident about their ability to deal with hazardous situations at the workplace [54]. On the other hand, this implies a lower awareness of safety management issues, which are considered at a less important level than daily operations and productivity. Such a finding is in line with the research insights of other studies investigating the safety attitude of workers in agriculture [33] as well as in other sectors [82].

Finally, it is worth mentioning that the *t*-test analyses brought to light a statistically significant correlation in the responses only of the comparison made between managers and workers, while the other comparisons concerning the male-female and young-elder subgroups indicated the absence of statistically significant differences. On the one hand, this could be explained by the assumption that managers and workers have a different level of expertise, while such a characteristic cannot be taken into account in the other subgroups. On the other hand, the nature of the questionnaire itself is mostly aimed at bringing to light shared safety perceptions among organizational units [66], which can seldom be found in small-sized companies as those in our case study.

### 5.2. Research Implications

Based on the above considerations, some general assumptions can be made to pinpoint the study findings. Firstly, the results achieved show a common low perception of safety issues in the sector. Given that in the considered sample farmers were involved in different agricultural activities, such an output can be considered transversal in the sector. In fact, unlike the majority of previous studies that investigated specific groups of farmers, in our case the results provide a more comprehensive snapshot of SC perception in agriculture. Moreover, the low level of perception of safety issues that emerged in this study is in line with the high rate of accidents that occur annually in the sector, validating the strong relationship between SC and occupational accidents, which has been documented by research in other types of industries [7,69,83].

Accordingly, this study can certainly contribute to augment knowledge on the farmers’ shared perceptions of management and workgroup safety policies, procedures, practices and behaviors [10], highlighting in particular the critical role of farmers’ risk-taking attitude and safety prioritization in their working tasks. These insights support the research findings by Caffaro and colleagues [84,85], who fostered the need to augment the learning of safety practices and safe behaviors among farmers. Similarly, the results also confirm the scarce inclination of farmers to adhere to safety protocols and guidelines, which has been brought to light by Irwin et al. [86].

Additionally, a different attitude toward safety issues has been registered based on both the position and gender of farmers. The different SC perception between managers and workers corroborated the evidence by Marin et al. [14] in the construction sector, showing that also in agriculture the SC perception is aligned with the role of the interviewed farmers. However, in our case, it emerged that managers in small farms have a SC perception more similar to that of field supervisors in the construction industry, since they share daily activities and the related risk exposure with workers. Therefore, in this case the gap claimed between “work as done” and “work as imagined” [87] is very slight.

Another interesting issue is related to the discrepancies that emerged based on gender. Although the results did not show a significance from the statistical point of view, this output can be considered a first attempt at screening occupational safety discrepancies between genders in agriculture. The descriptive output of this analysis indicated that women generally show a higher commitment to safety and care for each other’s safety, while they have a lower perception of safety management empowerment. To some extent, these findings reflect the research insights by Lin et al. [88] and could be used as a baseline for more detailed and larger investigations on the different safety attitudes between male and female workers in agriculture. For instance, the association of SC perception with the workload and specific job activity (e.g., tractor/machinery driver, manual harvester, pruner) might allow the definition of organizational measures that make the workers perceive a higher level of safety.

Finally, data based on the age of respondents confirm that a larger effort is needed in safety information and training of farmers: in particular, since it appears that younger farmers have a higher safety commitment and priority than aged co-workers, a major effort in safety information and training should be paid for the latter (i.e., workers ≥36 years old). In fact, although farmers in this subgroup certainly have a longer working experience, they mostly have a lower level of education and in accordance with accident statistics they are more vulnerable due to an underestimation of hazardous situations and a scarce attitude in following safety regulations properly (e.g., deviation from safety procedures when using tractors or other agricultural machinery) [89].

Overall, the main strength of this study relies on the fact that it is the first attempt to investigate SC perception of farmers by means of the NOSACQ-50 questionnaire, which is a validated assessment tool and whose results can be easily compared to the results of SC evaluations from other studies, augmenting knowledge on SC perception in agriculture. Despite the descriptive nature of the results, consistent with Mosly and Makki [16], they can be used to better understand the specific areas of improvement to enhance safety climate in small companies operating in the agricultural sector. Therefore, such an output can contribute to fostering research on social and workplace aspects of sustainable agricultural systems, given the natural synergy between sustainability on one side, and ergonomics and human factors on the other side [90,91,92].

### 5.3. Limitations

The main limit of this study is certainly represented by the sample size of the respondents. In fact, although the overall number of the interviewed farmers is in line with the range of 100–200 cases, which suits for NOSACQ-50 analyses [66], the further screening of results based on the position, gender, etc. within the sample can reduce the generalization of the aforementioned findings. Hence, on the one hand, it is clear that a larger sample of respondents could allow a more accurate understanding of their SC perception [93]. On the other hand, it has to be remarked that the number of complete responses collected is in line with the sampling size suggested in several studies concerning qualitative case study research aimed at generating new propositions and understandings [94,95].

Moreover, since this research is an exploratory descriptive study aimed at taking the first step in addressing the identified literature gaps, the generalization of the results is also limited by the focus on the companies’ typology and the specific regional context where the interviews were carried out.

Additionally, the analysis did not distinguish the different work profiles of the interviewed sample, i.e., the specific tasks they carry out daily. On the one hand, this aspect is difficult to determine since in small-sized companies’ farmers are usually engaged in different activities depending on the specific needs of the moment (i.e., tractor/machinery use, manual handling, pruning, etc.). Therefore, the same people carry out different tasks and use different types of work equipment. On the other hand, a more detailed analysis of working tasks could allow the identification of safety perceptions related to specific activities, providing more thorough information for the implementation of ad hoc safety measures at the company level such as instructions and training. Consequently, further studies along these lines are required in order to explore the correlation with specific personal and occupational factors, including those related to the farmers’ nationality. With reference to the latter aspect, it has to be underlined that in the present study the nationality of farmers was not considered because there were no foreigners in the selected sample.

## 6. Conclusions

Safety climate is described as the combination of shared perceptions among workers on the procedures, practices, attitudes, and behaviors related to occupational safety. Accordingly, it has been demonstrated that investigating SC perception can provide positive outcomes aimed not only at bringing to light precursors of accidents, but also to augment shared knowledge and awareness among workers and managers on safety behavior, safety practices and safety compliance.

However, although it is recognized as a very hazardous sector, very few studies have investigated safety climate among farmers and, in particular, to the authors’ knowledge, no studies have applied the NOSACQ-50 questionnaire for a cross-sectional analysis among farmers. Therefore, the main merit of this study consists in providing a first screening of SC among farmers by means of a cross-sectional application of NOSACQ-50, whose validity and reliability has been tested in various different sectors.

The results from this study show a low level of SC perception of farmers in general, bringing to light in particular their high risk-taking attitude and low safety prioritization. These findings are consistent with previous research on safety behavior and accident analyses in the agriculture sector. Moreover, screening SC perceptions separately from various levels (such as age, gender and position within the organization) provided a more thorough appraisal of this complex work environment.

Accordingly, the results of this study suggest that specific measures to improve SC in the analyzed context could be taken based on the following issues:

- A simplification of safety “red-tape” for this type of company could help managers to implement practical safety measures at the workplace more effectively. This can have positive effects on both the managers’ perception of their safety commitment and the workers’ perception of the attention paid by managers to safety issues. In particular, this could make the conformity to safety obligations easier especially for aged owners/managers.
- Practical safety information and training could help both managers and farmers to deal with hazardous situations more consciously, reducing their risk-taking attitude and behavior. This could be beneficial especially for elder farmers and those with a low level of education to better understand and comply with safe procedures in daily activities (e.g., the proper use of tractors and other machinery, the use of personal protective equipment, etc.).
- A rewarding-praising system related to safety issues could help both the safety communication system and the workers’ trust in the efficacy of safety management at the workplace.
- Sharing the definition of safety organizational measures of daily activities and workloads could reduce SC discrepancies based on gender and age, improving safety motivation and participation.

In conclusion, this study can represent a baseline for further human-centered investigations aimed at reducing the occurrence of accidents in agriculture by means of specific and tailor-made interventions both at company (e.g., training and information activities) and normative (e.g., safety best practices) levels. Thus, such an output can certainly contribute to the improvement of social and workplace aspects of sustainable agricultural systems.

## Figures and Tables

**Table 1:** Results of the literature review: the contributions are organized per year from 2020 to 2004.

Code	Author	Year	Objective
[43]	Arcury et al.	2020	The work describes a community-based investigation on the effects of farm work on the health and development of Latinx child farmworkers. Safety climate (SC) assessment is a part of the investigation, which also includes safety/risk attitude, and vulnerability as key factors of work safety culture.
[44]	Kongsvik et al.	2019	The study analyzes which factors can influence the reporting of hazardous events in fish farming activities by means of a questionnaire where individual factors, company factors and SC factors were included.
[45]	Grimbuhler and Viel	2019	The study investigates SC among vineyard workers (farm managers and pesticide operators) based on a specific SC questionnaire, which was focused on the use of best practices when dealing with pesticides.
[46]	Wilmsen et al.	2019	The authors investigate workplace organizational factors and SC among forestry workers who had self-reported medical treatment of and recovery from a work-related injury. Interviews included questions about safety training, interviewee’s experience in the forestry industry and demographics.
[47]	Ramos et al.	2019	The study investigates the problem of alcohol consumption among Latino migrant farmworkers, including the analysis of work SC as a predictor of alcohol consumption and negative consequences from alcohol use.
[35]	Irwin, A., Poots, J.	2018	The study analyzes the potential predictors of non-technical skills’ attitudes in farming, considering the following elements: individual traits, attitudes toward safety, and the presence of environmental stressors.
[48]	Swanberg et al.	2017	The study investigates the work factors associated with elevated musculoskeletal discomfort among Latino thoroughbred farm workers, including the measure of SC.
[49]	Tribble et al.	2016	This study considers a sample of Latino farmworkers and Latino manual non-farmworkers to compare mental health between them and determine whether differences in musculoskeletal disorders between them can be related to indicators of mental health and work organization.
[50]	Arcury et al.	2015	This study examines associations of work organization with health characteristics of immigrant women in farmworker families with the goal of describing the work organization for Latinas in farmworker families and defining the association of work organization with indicators of health.
[51]	Arcury et al.	2015	The study analyzes the importance of perceived work SC among temporary migrant farmworkers. SC perceptions were associated to job characteristics, job hazards, and stressors.
[52]	Kearney et al.	2015	Work SC among hired youth farmworkers is investigated in order to determine the association between work safety climate and occupational safety behaviors and injuries.
[53]	Arcury et al.	2015	This study investigates the associations between work safety culture and injuries among these farmworkers, where safety culture comprises behavioral, situational, and psychological elements. The investigation of the latter is carried out through a SC assessment.
[54]	Savadori et al.	2015	The study focuses on employees’ perception of SC and its influence on safety behavior. In particular, safety compliance and safety participation are considered to measure psychological SC.
[55]	Swanberg et al.	2013	This study focused on the investigation of those work organization factors that are associated with work-related illness, injury, and missed work due to work related illness or injury of Latino crop and horse workers.
[56]	Arcury et al.	2012	This study analyses migrant farmworkers’ work safety climate with the aim of determining the association of work SC with musculoskeletal discomfort, working while injured or ill, and depressive symptoms.
[57]	Cigularov et al.	2009	This study focuses on the analysis of SC among young farm workers to investigate the relationship between SC and work-related errors.
[4]	Seo et al.	2004	A 32-item questionnaire is developed based on literature review and experts judgement to assess SC among workers at grain elevator facilities.

**Table 2:** Criteria suggested by the National Research Centre for the Working Environment of Denmark for the interpretation of the results of the Nordic Safety Climate Questionnaire (NOSACQ-50) questionnaire (source: [39]).

Score (s)	Level	Meaning
s > 3.30	good	maintaining and continuing development of the SC dimension
3.00 < s < 3.30	fairly good	the SC dimension needs a certain improvement
2.70 < s < 2.99	fairly low	the SC dimension needs an improvement
s < 2.70	low	the SC dimension needs a great improvement

**Table 3:** Background information of the interviewed sample.

Information	Type	Values
Age (106 respondents)	Mean	43.9 years
	Max	72 years
	Min	20 years
Gender (108 respondents)	Male	68
	Female	40
Position (107 respondents)	Manager	37
	Worker	70

**Table 4:** Details of the scores of NOSACQ-50 divided per dimensions (mean values).

Dimensions	Total	Managers	Workers	Males	Females
Dim1	2.92	3.15	2.80	2.92	2.92
Dim2	2.99	3.29	2.93	3.02	2.95
Dim3	3.06	3.23	2.98	3.02	3.14
Dim4	2.84	2.91	2.79	2.80	2.92
Dim5	2.67	2.76	2.62	2.67	2.68
Dim6	3.02	3.07	2.99	3.00	3.06
Dim7	2.95	3.17	2.85	2.96	2.94

**Table 5:** Results of the *t*-test analyses, where *t* indicates the *t*-test output, while *p* indicates the level of significance (* indicates *p* < 0.05).

**Managers (Dataset 1)—Workers (Dataset 2)**														
	**Dim1**		**Dim2**		**Dim3**		**Dim4**		**Dim5**		**Dim6**		**Dim7**	
**Dataset**	1	2	1	2	1	2	1	2	1	2	1	2	1	2
**Sample size**	37	70	37	70	37	70	37	70	37	70	37	70	37	70
**Average value**	3.15	2.80	3.29	2.83	3.23	2.98	2.91	2.79	2.76	2.62	3.07	2.99	3.17	2.85
**Standard Deviation**	0.52	0.34	0.47	0.26	0.54	0.32	0.55	0.40	0.54	0.45	0.40	0.23	0.43	0.37
***t***	4.141		6.370		2.904		1.301		1.436		1.341		4.009	
***p***	0.00007 *		5.1 × 10^−9^ *		0.0045 *		0.1959		0.1538		0.1829		0.0001 *	
**Males (Dataset 1)—Females (Dataset 2)**														
	**Dim1**		**Dim2**		**Dim3**		**Dim4**		**Dim5**		**Dim6**		**Dim7**	
**Dataset**	1	2	1	2	1	2	1	2	1	2	1	2	1	2
**Sample size**	68	40	68	40	68	40	68	40	68	40	68	40	68	40
**Average value**	2.92	2.92	3.02	2.95	3.02	3.14	2.80	2.92	2.67	2.68	3.00	3.06	2.96	2.94
**Standard Deviation**	0.463	0.395	0.436	0.360	0.456	0.352	0.460	0.471	0.522	0.404	0.294	0.310	0.448	0.364
***t***	0.056		0.818		1.377		1.344		0.066		0.880		0.206	
***p***	0.955		0.415		0.171		0.182		0.948		0.381		0.837	
**20–35 Years Old (Dataset 1)—≥36 Years Old (Dataset 2)**														
	**Dim1**		**Dim2**		**Dim3**		**Dim4**		**Dim5**		**Dim6**		**Dim7**	
**Dataset**	1	2	1	2	1	2	1	2	1	2	1	2	1	2
**Sample size**	27	79	27	79	27	79	27	79	27	79	27	79	27	79
**Average value**	2.99	2.90	2.98	2.99	3.14	3.05	2.95	2.80	2.82	2.63	3.01	3.02	3.02	2.94
**Standard Deviation**	0.520	0.411	0.385	0.421	0.370	0.443	0.394	0.479	0.445	0.487	0.236	0.322	0.455	0.404
***t***	0.862		0.133		0.942		1.475		1.878		0.169		0.868	
***p***	0.390		0.894		0.348		0.143		0.063		0.866		0.387	
